# Exploring urinary incontinence in hospitalised older women: A mixed methods investigation of prevalence and nurse perspectives

**DOI:** 10.1177/17455057241295607

**Published:** 2024-12-15

**Authors:** Isobel McMillan, Liz Doxford-Hook, Julie Wood, Yu Fu, Linda McGowan, Heather Iles-Smith

**Affiliations:** 1 School of Health and Society, The University of Salford, Salford, UK; 2 Leeds Teaching Hospitals NHS Trust, Leeds, UK; 3 Salford Royal NHS Foundation Trust, Salford, UK; 4 Newcastle University, Newcastle upon Tyne, UK; 5 The University of Leeds, Leeds, UK

**Keywords:** incontinence of urine, hospital admission, older women, prevalence, mixed methods

## Abstract

**Background:**

Approximately 40% of older women in the community report experiencing urinary incontinence (UI); prevalence within secondary care is unknown. Illness, comorbidities, and hospital environments are likely to lead to higher prevalence.

**Objectives:**

This study aimed to establish UI prevalence in older women admitted to hospitals and understand the views and knowledge of ward nurses in relation to older women’s UI.

**Design:**

An explanatory mixed methods study was conducted including a retrospective study of women ⩾55 years admitted to a large NHS hospital and qualitative interviews with nurses to gain an understanding of views, knowledge and perceptions of women’s UI and related care.

**Method:**

UI prevalence was determined using the nursing assessment (elimination) and *International Classification of Diseases 10th Revision* (ICD-10) codes for women ⩾55 years admitted to the hospital (November 2019 to February 2020); continence and demographic electronic patient care records data were extracted. Twenty ward nurses participated in interviews to explore views, knowledge and perceptions of UI care.

**Results:**

11.0% (*n* = 631) of the cohort (5,757) were recorded as having UI. Nurse interviews revealed six themes: (1) Normalisation and misconceptions of UI: nurses believed UI could not be improved, (2) limited knowledge and training: nurses expressed limited UI knowledge and a training need, (3) pad culture: continence pad use was high, (4) barriers to care: staffing issues were expressed as problematic, (5) UI under-reporting: nurses only categorised women with complete UI and others as “having an accident”, (6) catheter use in relation to UI: catheters were reported as a last resort.

**Conclusion:**

As community UI prevalence is 40%, our results (11%) suggest that UI is being underreported. Qualitative findings suggest that nurses have limited knowledge and training on continence care and under-report based on UI misconceptions. Our results suggest that ward nurses require dedicated UI training based on older women’s needs.

## Introduction

Approximately 40% of older (⩾55 years) women report having trouble with their urinary continence in their daily life.^1^ While urinary incontinence (UI) exists as an issue for both sexes the physiological differences between the sexes and changes post-menopause can result in specific issues that can impact urinary continence for older women, including genitourinary syndrome of menopause,^2^ vaginal prolapse and pelvic floor dysfunction (often due to childbirth).^3^

UI can manifest in different ways and to different degrees of severity, for example UI that occurs due to increased physical pressure (e.g. sneezing, coughing) is known as stress UI, whereas a sudden/uncomfortable urge to urinate is known as urgency UI and a combination of the two types is known as mixed UI.^4^

Women’s lives can be significantly impacted by UI including reduced quality of life, psychological health and confidence, as well as feelings of reduced sexuality and societal exclusion.^5^ Some women report feeling embarrassment and a sense of taboo related to their continence issues and may not seek help or discuss the matter with others including their partners.^5–8^ Older women are also less likely to be referred to continence care by their General Practitioner.^9^

Despite its high prevalence in older women, studies have revealed that women’s knowledge of UI is poor.^10–12^ These studies have demonstrated both poor knowledge and potentially harmful misconceptions, particularly in relation to risk, prevention, treatment, and management options. Potential opportunities to increase women’s knowledge of UI and management options should therefore be capitalised on.

The majority of empirical UI research has been conducted in the community rather than hospital in-patient settings. The presence of comorbidities and illness leading to hospitalisation, as well as factors related to the hospital environment, would suggest a higher prevalence rate of UI for older women within hospitals compared to community settings (40%).^1^ However, our scoping review^13^ revealed a dearth of empirical research related to older women’s UI during hospital admission. Therefore, there is a need for a greater understanding of the prevalence and incidence of women (⩾55 years) who experience UI during hospital admission.

One way to explore this is through the use of electronic patient care records (EPCR). These records include nursing assessments, based on the activities of daily living^14^ and include a person’s continence status. Assessments are routinely undertaken on admission and repeated either weekly or when the patients’ health condition, clinical status or circumstances change (i.e. post-surgery or unplanned clinical event occurs). Using EPCR is therefore a potentially useful means to better understand the prevalence and incidence of UI in older women as reported by nurses to provide greater insight.

There is also a need to gain an understanding of ward-based nurse’s (inclusive of the wider nursing team) views, knowledge and perceptions of UI, and how they care for women who experience UI during an in-patient admission. Given their contact with women during hospital admissions, nurses could provide a perfect opportunity to educate women about their UI and potential management options that are available to them.

The following paper describes a mixed-method study to determine the prevalence and incidence of UI for older women (⩾55 years) during hospital admission and an exploration of nurses’ views, knowledge and perceptions of providing care for older women with UI during hospital admission. This was part of a wider study (see full protocol^15^), which additionally aimed to determine the health-related risk factors and mortality rates associated with UI, for older women admitted to the hospital; these results will be reported separately.

## Method

This was an explanatory-sequential mixed-method study^16^ where the quantitative data were collected and analysed first, and informed the qualitative data collection and analysis. Qualitative data were used to explain the quantitative data. There were therefore three phases:

- Phase 1: Assessing prevalence and incidence of UI using EPCR
- Phase 2: Qualitative interviews with nurses
- Phase 3: Integration and interpretation of data

The methodology for each phase is discussed below and the results for each phase are reported separately, with integration and interpretation of findings within the discussion section. The Good Reporting of A Mixed Methods Study (GRAMMS) Guidelines^17^ were followed when preparing this manuscript.

### Phase 1: assessing prevalence and incidence of UI using EPCR

This phase of the study was a retrospective study of women aged 55 years or over who were admitted to a large NHS hospital in the north of England, between 1 November 2019 and 29 February 2020. Electronic in-patient data were used, including medical diagnoses and nursing assessment to determine the prevalence of UI on admission, and incidence of women (⩾55 years) becoming incontinent of urine (new cases over time) during admission to the hospital.

The nursing assessment is an assessment conducted verbally by nurses that all patients receive on admission to hospital. The assessment is based on the activities of daily living^18^ and recorded on the patient’s EPCR. This nursing assessment is a simple assessment involving asking the patient whether they normally and currently experience any issues with bowel movements or passing urine. Each patient is recorded as either ‘continent / no problems’, ‘incontinent of urine’, or ‘catheter’ for the ‘elimination bladder’ part of the assessment and as either ‘continent / no problems’, ‘incontinent of faeces’, or ‘stoma’ on the ‘elimination bowel’ part of the assessment.

Medical diagnosis of UI was gained by looking at the assigned *International Classification of Diseases 10th Revision* (ICD-10) codes: N39.3, N39.4 and R32.X.

#### Data extraction

De-identified EPCR data were extracted from the hospital data warehouse. Women who had withdrawn consent for the use of their electronic health records for research and were registered with the NHS Digital national data opt-out service, were excluded from the analysis. University of Salford Ethics and NHS Health Research Authority approval for the research was granted (IRAS project ID: 303118).

The de-identified data were securely transferred to researchers at the University of Salford using secure (encrypted) NHS mail in line with NHS digital guidance (https://digital.nhs.uk/services/nhsmail/guidance-for-sending-secure-email).

Data were included for all women ⩾55 years. This age group was selected as they have been identified to be more likely to experience UI than women under 55 years.^19–21^ Full list of inclusion and exclusion criteria is listed in Table 1.

**Table 1.** Inclusion and exclusion criteria.

Criteria	Included	Excluded
Admission type	Individuals with an in-patient admission	Day cases with no overnight stay
Sex	Female	Male
Age	55 years and over at the time of admission	Under 55 years
Reason for admission		Individuals admitted for a surgical procedure directly related to UI (for stress and urgency UI)^a^
Data access		Individuals who had registered through the National data opt-out (NHS Digital, 2021)

MUS: midurethral slings; UI: urinary incontinence.

a Stress incontinence surgery includes traditional sling, colposuspension, MUS (retropubic or transobtuator), single incision slings, bladder neck needle suspension or anterior repair.^22^ Urgency UI surgery includes augmentation cystoplasty, urinary diversion.^23^

Power analysis for sample size calculation has also been reported in our protocol paper.^15^ We used the following formula to estimate the sample size needed to achieve sufficient accuracy in estimating prevalence:

$$n=\frac{Z_{\alpha }^{2}p(1-p)}{L^{2}}$$

where *Z*α is the two-tailed *Z*-value from the confidence level, *p* is the expected proportion of patients that will be recorded as having UI, and *L* is the precision on the expected proportion (see SRUC: Epidemiology Resources sample size estimator app https://epidemiology.sruc.ac.uk/shiny/apps/samplesize/). Based on literature describing community-based prevalence we estimated a prevalence of 50%. Based on this, the sample size needed was 2,401 in order to have 95% confidence that the ‘true’ prevalence of incontinence is within 2 percentage points of this (i.e. prevalence between 48% and 52%).

#### Data analysis

Data were separated into four cohorts^15^:

1. Continent: This group included all individuals who were recorded as ‘continent’ on all nursing assessments during admission. Individuals with any ‘incontinent’ ICD-10 codes or individuals recorded as having a catheter on any nursing assessment were excluded from this group.
2. UI: This group included individuals recorded as incontinent of urine in any nursing assessment during admission and individuals with a UI ICD-10 code in medical diagnosis during admission. Individuals who were also recorded as having faecal incontinence on any nursing assessment or individuals recorded as having a catheter on any nursing assessment were excluded from this group.
3. Double incontinent: This group included individuals recorded as incontinent of both urine and faeces on nursing assessments. Individuals recorded as having a catheter on any nursing assessment were excluded from this group.
4. Indwelling catheter: This group included individuals recorded as having a catheter on any nursing assessment.

The cohorts were used to determine prevalence and incidence (Iles-Smith et.al., 2023). Prevalence was established through the count and percentage of women falling within the four continence cohorts: (1) continent, (2) UI, (3) double incontinent, and (4) those with an indwelling catheter. Incidence of women (>55 years) becoming incontinent of urine during admission was determined through counts and percentages over time.

### Phase 2: qualitative interviews with nursing staff

This phase of the study used qualitative interviews to gain an understanding of nurse’s views, knowledge and perceptions of providing care for older women with UI during hospital admission. Their use of nursing assessments was also explored. Some of the questions asked during the interviews were informed by the quantitative findings from phase 1. For pragmatic reasons, nurses included in the interviews were employed at a different hospital to the hospital providing EPCR data for phase 1.

#### Participant selection and data collection

Semi-structured interviews were conducted with 20 nurses working on a variety of female or mixed in-patient wards at a large, northern, NHS tertiary hospital. Interviews were with those from the wider nursing team both registered (UK Nursing and Midwifery Council) and non-registered nurses.

To recruit participants, ward managers were contacted, by the researchers, and asked for permission to attend wards to discuss the study with staff; both written and verbal study information was provided to potential participants. Informed consent was taken prior to interviews taking place. Interviews were conducted either over the phone, on Microsoft teams or face to face, depending on participant preference and lasted approximately 30 min. Recruitment of participants continued until data saturation was reached. The interview schedule (see Supplemental Material) was informed by both the literature and findings from the first phase of this study.

#### Data analysis

All interviews were audio recorded and transcribed verbatim. The Framework approach,^24^ along with thematic analysis including induction and deduction and using the principles of Braun and Clark,^25^ was used during the analysis. Framework analysis is underpinned with five interconnected stages including, familiarisation, identifying a thematic framework, indexing, charting, mapping and interpreting. Analysis involved a series of interconnected stages enabling the researcher to move back and forth across the data until a coherent account emerges.^24^

### Phase 3: integration and interpretation of data

As this study followed an explanatory-sequential design the quantitative findings from phase 1 were used to inform the qualitative interviews in phase 2. The qualitative results were used to provide context and deeper understanding of the observed quantitative results. In phase 3 the findings from each of the two phases were therefore interpreted and synthesised to draw overall conclusions. Interpretation and synthesis of the data were led by the first author (IM) with input from other researchers (authors HIS, LDH, YF and LM) to ensure rigour.

## Results

### Phase 1: prevalence and incidence of UI

#### Cohort

**Figure 1.**
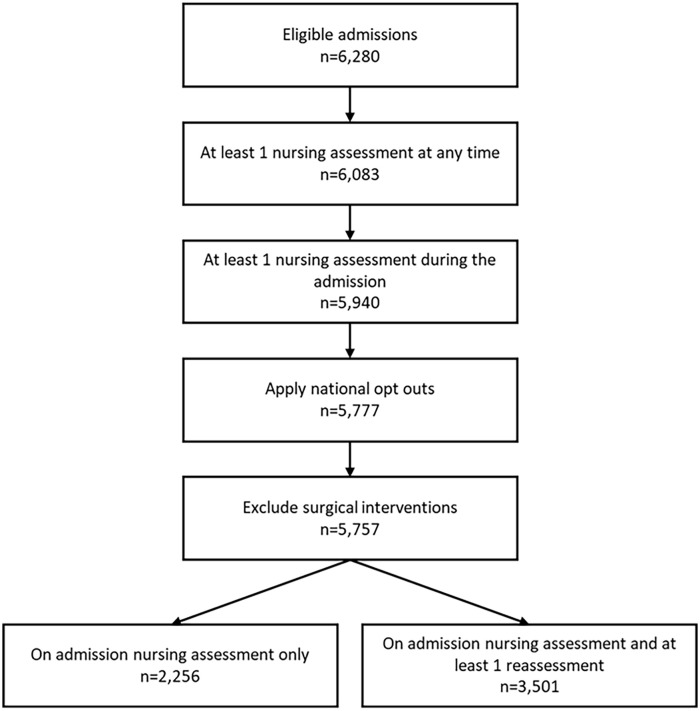
Total of individuals in the cohort.

Data were available for 5,940 women ⩾55 years who had at least one nursing assessment. Of these, 163 were registered with the NHS Digital national data opt-out service and therefore were not included. A further 20 women were excluded due to having a surgical intervention for UI, leaving a total cohort of 5,757 (see Figure 1).

Of the total cohort, 2,256 (39.1%) only had one nursing assessment which was on admission. The remaining cohort (*n* = 3,501; 60.6%) had at least one nursing reassessment during their admission. These reassessments were used to calculate incidence estimates for UI. The average number of assessments that an individual received was 2.57, and the average length of stay in hospital was 10.26 days. Figure 1 shows the total number of eligible individuals (6,280) for the cohort. Of the total eligible individuals, 6,083 (96.9%) have at least one nursing assessment recorded at any time (see Figure 1).

#### Prevalence and incidence

**Figure 2.**
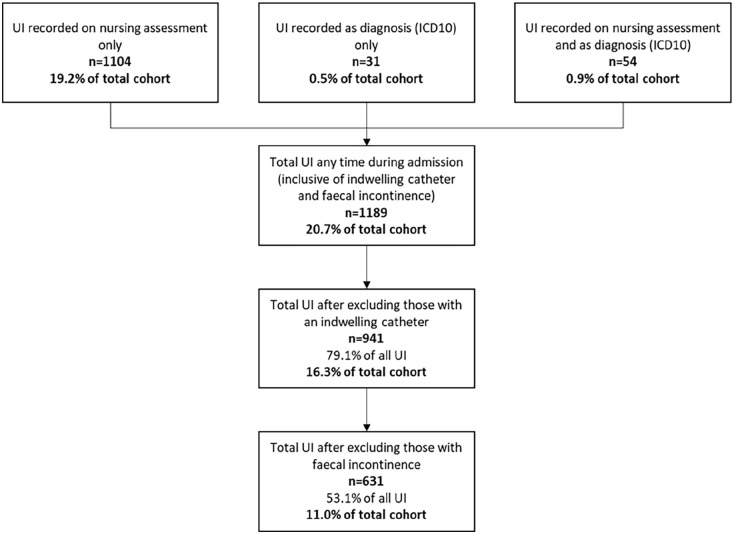
Breakdown of individuals classified as UI. UI: urinary incontinence.

The total number of women recorded with UI either as a diagnosis (using ICD-10 codes) or recorded as ‘incontinent of urine’ on the nursing assessment at any time during admission was *n* = 1,189 (20.7%). Prevalence was 16.3% when those with an indwelling catheter (*n* = 248) were removed from the total cohort, and when those with faecal incontinence (*n* = 310) were removed only 11.0% of the total were classified as having UI (Figure 2). The majority of individuals in the UI group were categorised by the nursing assessment (see Figure 2).

Excluding individuals with catheters and double incontinence, only 47 individuals had UI ICD-10 codes recorded in ‘any diagnosis’ variable during their admission. This compares with 608 individuals recorded as UI on any nursing assessment.

**Figure 3.**
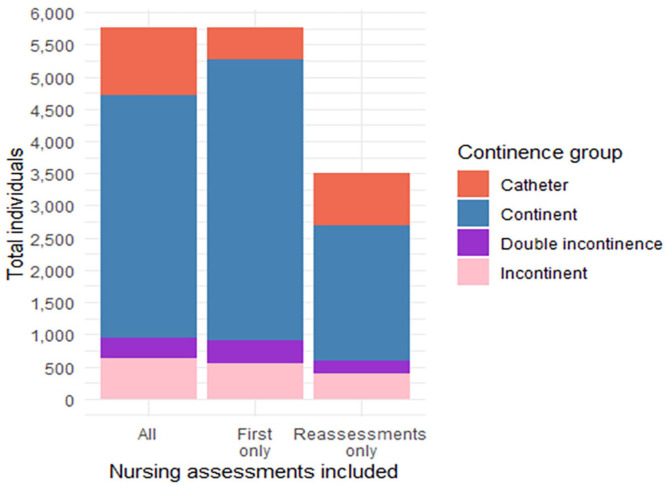
Total of individuals in each continence group using all, first and reassessment nursing assessments.

Comparison of the assignment of continence groups depending on which nursing assessments were used is seen in Table 2 and Figure 3. The percentages are similar for the UI group when including all assessments (first nursing assessment and reassessment). There was decreased prevalence within the continent group and an increase in the catheter group from first assessment to reassessment.

**Table 2.** Total of individuals in each continence group using diagnosis codes and all, first and reassessment nursing assessments.

Cohort group	All nursing assessments		First nursing assessments only		Nursing reassessments only	
	*n*	%	*n*	%	*n*	%
UI^a^	631	11.0	548	9.5	396	11.3
Double incontinence	310	5.4	361	6.3	198	5.7
Catheter	1,037	18.0	496	8.6	813	23.2
Continent	3,779	65.6	4,352	75.6	2,094	59.8
Total	5,757	100.0	5,757	100.0	3,501	100.0

UI: urinary incontinence; ICD-10: *International Classification of Diseases 10th Revision*.

a Includes urinary incontinent ICD-10 diagnosis codes.

Of the total who were ‘continent’ at the first nursing assessment (*n* = 4,352), 2,509 had a reassessment. Of these reassessments, excluding individuals with a catheter or those with double incontinence, 166 (6.6%) had an ‘incontinent of urine’ status at nursing reassessment.

### Phase 2: qualitative interviews with nursing staff

Twenty nursing staff were interviewed (Table 3 summarises participant characteristics). Six overarching themes emerged from the interviews, (1) Normalisation and misconceptions of UI, (2) limited knowledge and training, (3) pad culture, (4) barriers to care, (5) UI under-reporting, (6) catheter use in relation to UI. These themes and their associated sub-themes are summarised in Table 4 along with example quotes for each.

**Table 3.** Participant characteristics.

Characteristic category	*N*	%
Sex		
Female	15	75
Male	5	25
Age		
18–25	1	5
26–35	9	45
36–45	4	20
46–55	4	20
56+	2	10
Ethnicity		
White British	16	80
Asian/British Asian	3	15
Black/African/Caribbean/British Black	1	5
Job role		
Staff nurse	7	35
Clinical support worker	9	45
Sister	2	10
Ward manager	1	5
Nurse team lead	1	5
Ward specialty		
Elective surgery	4	20
General medical	1	5
Intermediate care	3	15
Gynaecology	1	5
Ageing and complex medicine	5	25
Neurology	4	20
Renal	1	5
General surgery	1	5

**Table 4.** Themes and example quotes.

Main themes	Sub-themes	Quotes
Theme 1: Normalisation and misconceptions of UI	Normalisation of UI in older women	1. ‘I think we have a less dignified situation, being female, to be honest and I do feel sorry for many of them, the fact that they’re. . . Yes, it just seems to be looked as a natural progression and that’s that!’ (Participant 4) 2. ‘It’s just expected really, isn’t it? In a lot of occasions, you do just expect it to do with their age’. (Participant 8)
	Misconceptions of urgency UI	3. ‘I stress to patients is if you need the toilet and you can’t get there press the buzzer straight away, don’t wait till your absolutely desperate’. (Participant 10) 4. ‘She said to me, “I couldn’t get there in time”. So that’s what I did notice, and I said, “Why didn’t you ring for me, and I would have taken you, got you a chair and wheeled you quickly to the toilet”’. (Participant 14) 5. ‘So I think that’s more of, I don’t know, maybe you’re not holding it’. (Participant 12)
	Understanding of patient experience	6. ‘Quite embarrassing to be honest because even if it was me, I’d feel embarrassed. I know it’s obviously normal to have it and it’s quite common but yes, a lot of people feel embarrassed about it and they apologise a lot when you do clean them and I do feel bad for them sometimes’. (Participant 13) 7. ‘It can be embarrassing for them, especially if they’ve never had this issue before and they’ve been urine independent for a long time. You’ve just got to reassure them, let them know that we see this on a daily basis and it’s nothing to be embarrassed about, it is one of those things that can happen. It can be uncomfortable for them but as long as you know you’re keeping their dignity, making them feel comfortable’. (Participant 15)
	Having an accident	8. ‘It’s just classed as an accident if they do. You just reassure them that it was an accident’. (Participant 8)
Theme 2: Limited knowledge and training	Expressed need for training	9. ‘To be honest I don’t recall ever receiving any particular training on it. Whether I did have a quick whistle-stop tour of it when I was newly qualified or a student, but I don’t recall having any training on it’. (Participant 1) 10. ‘No. The only training that I’ve had is the removing the catheters, that’s all’. (Participant 3) 11. ‘I think it is a big part of nursing care and I think obviously if there’s anything more we can be doing to support our patients, or do it in a more effective way, or a better way for them, then I think obviously we’d welcome that training, yes’. (Participant 1)
	Causes	12. ‘Yes. It is an age thing, I think, as you get older. Just everything goes south, doesn’t it?!’. (Participant 3) 13. ‘Interviewer: What do you think causes urinary incontinence? 014: Well, is it not old age? I think it’s old age, but I mean, it’s something, isn’t it? Well, I think if they’ve got dementia, that would cause it, because they won’t even know, will they’. (Participant 14) 14. ‘Sometimes your patients that have got dementia, and things like that where they might forget, or it’s that they know they’re doing it, but they can’t articulate it fast enough and get bed pan fast enough’. (Participant 1)
	Interventions	15. ‘So the knowledge is not really taught us to be honest. It’s not. You just say, “They’re incontinent, give them pad”’. (Participant 8) 16. ‘So it’s best just to react to the incontinence rather than try and prevent it, because incontinence isn’t a medical issue really. That’s just an accident’. (Participant 19)
Theme 3: Pad culture	Over-reliance on pads	17. ‘Then, sometimes I’ve noticed when they go in hospital, the number of times the hospital makes them incontinent by putting pads on and things like that because they haven’t got time to take people to the toilet because it takes longer. It’s quicker to just put a pad on a change a pad. We’ve had that a few times where they don’t want to lose the pad because they’ve had it on in hospital for weeks’. (Participant 5) 18. ‘Yes, just stick a pad on them, rather than offering other alternatives, such as walking them to the bathroom. I feel like some staff just think it’s going to save me a bit of time, rather than changing the full bed, I’ll just stick this pad under it and hope it absorbs’. (Participant 7)
	Unsuitable products	19. ‘I can’t imagine they’re very comfortable. Sometimes I think if you’ve got a patient that’s bigger, obviously they (pads) can dig into the skin and things like that, so they’re not ideal’. (Participant 1) 20. ‘It’s really difficult with the styles of pads because not everything fits very well, do they? They move about in bed, you know, you place it and even if you use the net knickers, they get twisted, they’re quite stiff, so I do think that can be an issue’. (Participant 6)
	Terminology	21. ‘If the patients are dementia, we normally give her nappies’ (Participant 2) 22. ‘If they’ve got the full nappy on, it still possibly would seep through but it would be less and it would be more contained back into the crystals away from the skin’. (Participant 6)
Theme 4: Barriers to care	Staffing issues	23. ‘When you have a hectic schedule it’s quite busy. So we are not getting that much of time to, like, maintain the same level of things always. Yes, but we are doing our best’. (Participant 11) 24. ‘There’s only (so) much you can do, so if you’ve got five of them that needs to go on a bedpan, for example, we can’t assist five people at the same time. So, you see they’re sat down for a long time or they’re lying in bed and they’re lying in the wet, and it’s not something good for them. Not at all’. (Participant 17) 25. ‘Then, I think the other thing is the continence team take that long sometimes to come out that it’s a bit late sometimes if that makes sense. They’ve been incontinent for a while and they just carry on as they were because it’s took weeks and weeks and they’ve just got used to weeing in a pad, and even though they don’t like it, they feel that that’s just their lot. Sometimes I think the continence service itself, because of the pressure they’re under, will just give. . . I’ve noticed they just give very basic advice to some people and sort of, “We’ll give you – supply your pads. What pads do you want?” and that’s it sometimes without investigating why it’s happening and, “Is there a reason?”’. (Participant 5)
	Taboo subject	26. ‘Yes. So the female patients, as I already told you, they are much more embarrassed, sometimes, to tell you that they are incontinent or they can’t tell you when they are passing the urine or stools. So at that time, we will pitch our care support workers or any other of the nursing staff who is female around that’. (Participant 11) 27. ‘I said, “Do you want me to change you”, she said, “No, I want to do it myself”, she wanted to do it herself so I couldn’t get involved in anything like that, but I think, she was devastated, she said to me. Do you know, that’s hard for them really, isn’t it, when they’re independent like that’. (Participant 14)
Theme 5: UI under-reporting	Mismatch between perceived prevalence and reporting	28. ‘Yes, everyday, a big, quite a large number of patients are incontinent and obviously when I was at the care home most people were incontinent’. (Participant 8)
	UI classification	29. ‘You know what I mean, because she might get incontinent, but she’s not really incontinent’. (Participant 14) 30. ‘Yes, that’s a difficult one. I think as well, if they’ve got capacity and they can explain to you if they’ve tried to make it to the toilet then we wouldn’t’ (categorise as incontinent). (Participant 15)
	Use of nursing assessments	31. ‘I don’t ask sometimes the younger one. I always ask the elderly one’. (Participant 2) 32. ‘Interviewer: In terms of when you’re conducting nursing assessments, do you routinely ask all patients about urinary incontinence? 020: Not all, people that have like history of incontinence or more the elder, we ask’. (Participant 20) 33. ‘Interviewer: Do you think they’d redo an assessment if only continence changed? 007: I highly doubt it, no’. (Participant 7) 34. ‘Interviewer: What would trigger you to redo the nursing assessment? 006: I don’t, if I’m honest, because once you’ve done the nursing assessment, nobody looks back to it and that’s where I go back to that – so when you’ve done [Evolve?] and you fill it in, the joke has been that I’ve never known anyone to actually revert back to it, to look at any of it’. (Participant 6)
Theme 6: Catheter use in relation to UI	Last resort for skin integrity	35. ‘Sometimes if the patients have, has skin damage, we normally put catheter with them, yes, we normally put catheter with them because it’s very difficult to control the skin damage if it’s constant incontinence, it’s very difficult, so that’s why we put catheter with them’. (Participant 2)
	Removal on discharge	36. ‘We’re taking them out to basically do a trial without for discharge purposes because they weren’t actually catheterised before they came into hospital’. (Participant 5)
	Lack of patient advice on removal	37. ‘I would probably just obviously tell them about, just say, “Let us know when you want the toilet”, and we would monitor it. They’ve got a call bell, so they know they can press the call bell’. (Participant 1)

UI: urinary incontinence.

#### Theme 1: normalisation and misconceptions of UI

Normalisation of UI, particularly in older/elderly female patients was very apparent in interviews with all nursing staff. Participants felt that UI is an inevitable part of ageing and that there are few or no interventions to help or treat UI other than the use of continence pads (see quotes 1–2, Table 4). Nursing staff also had several other misconceptions about UI, for example there appeared to be a lack of understanding of urgency UI with some participants expressing frustration that women ‘didn’t ask soon enough’ or ‘weren’t holding it’ (see quotes 3–5, Table 4). Interestingly nurses also appeared to make clear distinctions between complete incontinence, and what they referred to as an ‘accident’. Nurses tended to define women who experienced incontinence but were able to ask for the toilet or understand that they had wet themselves, as having accidents and not experiencing UI (see quote 8, Table 4). This will be discussed further under the UI under-reporting theme.

#### Theme 2: limited knowledge and training

Most nurses interviewed had limited knowledge and had received no formal training related to UI, with the exception of catheterisation (see quotes 9–11, Table 4). They also specifically lacked understanding related to the potential causes and management options for UI. In terms of causes, most participants referenced only older age or neurocognitive issues (see quotes 12–14, Table 4). This links to both the normalisation of UI for older women, and also the misconception that UI is associated with those who are unable to ask for the toilet due to communication or cognition issues. Interviewees also demonstrated a lack of understanding or awareness of management options for UI outside of the use of continence pads. While some discussed ward-based toileting options, such as use of bedpans and commodes, continence pads were clearly the most used method for UI management on the wards (see quotes 15–16, Table 4). Management of UI for patients once they leave the ward tended not to be mentioned by interviewees, and only six participants made any reference to referring patients to specialist services (such as the community continence service or urogynaecology) to gain support with their incontinence. Self-management options also appeared to be an area of limited knowledge, with most nurses expressing they had no knowledge of self-management options (such as pelvic floor exercises, encouraging hydration, minimising constipation, or reducing caffeine intake). A few nurses did express that they had some knowledge of pelvic floor exercises, although this was through personal experience rather than training. All participants expressed the need for more training in continence care.

#### Theme 3: pad culture

The most commonly referenced method for managing UI on wards was continence pads. Interviews revealed that some nurses saw continence pads as the only real option for UI management. Some felt pads were being overused in some situations due to them being seen as easier than regularly toileting (see quotes 17–18, Table 4). Despite this, many interviewees expressed dissatisfaction with the suitability of continence pads available on wards often referring to them as ‘nappies’ due to the large size (see quotes 21–22, Table 4). Participants expressed a need for different sizes to offer patients, stating they felt pads did not help with pressure ulcers or infections (quotes 19–20, Table 4). A number of participants referenced women using their own continence products rather than the available products. Availability of different sizes and types of continence pads appeared to vary across wards and departments.

#### Theme 4: barriers to care

When discussing barriers to care most interviewees talked about reduced staffing and/or under-resourcing. Participants reported that staffing issues reduced the available time to attend to patients; impacting their ability to help with toileting and checking/changing continence pads as regularly as needed (see quotes 23–24, Table 4). Some nurses interviewed also referenced understaffing and underfunding of continence services resulting in long waiting times for women referred for support (see quote 25, Table 4). Another potential barrier to care expressed by some nurses was despite its prevalence, UI is seen as a taboo subject or a subject that female patients do not feel comfortable discussing. In some cases, participants discussed patients hiding the fact they experience UI from nurses and sometimes refusing help from staff (see quotes 26–27, Table 4).

#### Theme 5: UI under-reporting

When asked about the frequency at which they see patients with UI on the wards, most participants stated that it was a common occurrence in older female patients (see quotes 28–29, Table 4). The only exceptions to this were two nurses who both worked within elective surgery wards who stated that the demographics of patients that they typically see do not tend to experience UI. Despite discussing high frequency of UI in patients, when asked about nursing assessments and when a patient would be classified as incontinent of urine, most participants stated that they would not classify a person as incontinent of urine if they just had what they deemed as ‘accidents’. When probed further this belief appeared to be linked to the patient’s mental capacity and/or ability to communicate their toilet needs; those who could ask for the toilet but didn’t make it were deemed as having an accident and those who were unable to ask for the toilet were deemed incontinent of urine (see quotes 29–30, Table 4). When asked about undertaking the elimination (or continence) part of the nursing assessment, participant responses were mixed regarding whether they would ask all patients about their continence status. Some stated they would always ask all patients and others acknowledged they might only ask older patients (see quotes 31–32, Table 4). Several of the nurses interviewed also stated that if a patient’s continence status changed or they became aware of UI after the initial assessment, they were unlikely to redo the assessment to reflect the changes (see quote 33, Table 4). Some participants also commented on nursing assessments more generally as often not being used as part of care delivery or re-visited by nurses once completed (see quote 34, Table 4).

#### Theme 6: catheter use in relation to UI

When discussing catheter use in relation to UI the majority of interviewees stated that catheters would only be used as a last resort when women were at risk of developing pressure ulcers (see quote 35, Table 4). When asked about catheter removal, the majority of participants stated that the main reason a catheter would be removed was because the patient was due to be discharged. However, most participants stated that little direct advice or post-catheter removal care was given to patients, other than observing frequency of urination (see quote 36–37, Table 4).

### Phase 3: integration and interpretation of data

The combined findings from phases 1 and 2 of this study suggest that clinical teams are under-reporting UI in older women within both the ICD-10 coding and the nursing assessments. The prevalence and incidence results highlighted that only 11% of older women were recorded as experiencing UI alone in nursing assessments (20.6% inclusive of those with catheters and faecal incontinence) even though during qualitative interviews nurses reported that UI was a frequent occurrence on wards, particularly among older female patients. The qualitative interview findings suggest that this disparity between nurse’s observation of UI prevalence and what they are reporting on the nursing assessment may be due to nurses only classifying patients as incontinent of urine on the nursing assessment if they were completely unable to control their bladder or were unable to verbalise that they needed assistance. In addition to this, the interviews suggest that nursing assessments may not always be repeated when continence status changes. Participants also reported having limited knowledge and training in continence care and appeared to normalise UI, particularly in older women.

## Discussion

This study reports for the first time the prevalence (11%) of UI for older women admitted to hospital. The use of real-world data inclusive of electronic nursing assessments and ICD-10 codes provides insight into UK secondary care practice and culture, related to older women’s urinary continence. Given that 40% of older women living in the community report having issues with urinary continence,^1^ it was anticipated that our prevalence findings would be higher for women admitted to a large tertiary NHS hospital, due to the likely presence of co-morbid conditions, illness status and change of environment. However, results instead showed a much-reduced prevalence for female in-patients than expected. In particular, the recording of UI by medical staff with the use of ICD-10 codes, is almost non-existent within women’s EPCR, with only 47 of the 5,757 women being recorded as incontinent of urine. Similarly, but to a lesser degree, nursing practice related to recording UI through the elimination aspect of the nursing assessment also appears to under-report UI in the older female population. The likelihood of under-reporting by nurses through the nursing assessment is supported by the qualitative interview findings, which highlights the high frequency that the nurses came into contact with older women who experience UI, when delivering their care. This was described by almost all participants as a very regular occurrence.

The low levels of reporting could be due to a culture of normalisation of older women’s UI by clinicians and practitioners. This is suggested within the qualitative study findings where nursing staff describe UI being part of ageing for women and almost expected for those with dementia or reduced cognition. Nurses also had a tendency not to categorise UI for all patients experiencing UI. This appeared to be for several reasons, firstly many of the participants appeared to only classify women as experiencing incontinence of urine if they were completely unable to control their bladder. They instead referred to incontinence outside of this as accident. Secondly, several nurses stated that not all patients would be asked about urinary continence status when conducting nursing assessments and that they did not think nursing assessments would be updated if continence status changed. Our group previously reported the findings in a qualitative study of older women (55 years and above) that women themselves considered UI to be a normal part of ageing and that it was a ‘fact of life’.^26^ As none of the nurses in our study had received any continence training, beyond catheterisation, it is likely that cultural stereotypes or personal experiences play a part in their beliefs that UI is normal for older women.

Some nurses also felt that nursing assessments more generally were not used or re-visited once completed. This is an interesting finding as it calls into question how nursing assessments are conducted and used more generally. Further research on a wider scale into how EPCR and nursing assessments are used in care settings could be an area to explore, particularly as this data could be a useful future means to better understand a patient care and a means to explore the prevalence and incidence of a condition.

Our study also suggested an increased incidence of catheterisation during women’s admission, suggesting a potential cultural response by clinicians to UI as this increased from 8.6% catheterisation on admission to 23.2% at reassessment. When asked about catheterisation in interviews nurses stated that the main reason for insertion was for prevention of pressure ulcers. However, the interviews revealed limited understanding or description of the discomfort and complications of catheterisation such as infection, sexual dysfunction and reduced quality of life.^27^ Additionally, catheters were often reported as not being removed until discharge, which limits the opportunity for nursing staff (or women) to assess whether normal micturition has resumed. Nursing staff also stated that very limited advice is given to women on removal of a catheter. Nurses did not describe the use of catheter assessment tools or other important measures to reduce or identify urinary catheter-related infections, which can cause women pain, discomfort and lasting implications.

Interviews with nurses also revealed that nursing staff normalised UI, particularly for older women, and had misconceptions about UI, particularly in relation to urgency UI, where nurses did not demonstrate an understanding that the need to urinate can come on suddenly and urgently. Nurses interviewed also showed a lack of knowledge in terms of causes and potential interventions. These findings are consistent with studies that show poor knowledge of UI in the general population of women.^10–12^ They also demonstrate a clear need for training for nurses around continence and continence care. All nurses interviewed felt there was a lack of training given on continence and continence care and stated that they would welcome training in this area. To improve this deficit in knowledge, development of a training package for nurses should be considered for improving continence care within secondary care settings.

Another key finding from our study was the ‘pad culture’; an over-reliance on continence pads to manage UI onwards. This finding is consistent with an ethnographic study which identified a ‘culture of pad usage’, irrespective of continence status, to prevent leakage and safeguard episodes of UI, for dementia patients admitted to secondary care. This study also reported an over-reliance on pads which led to an increased incidence of UI.^28^ While staffing issues may be cited as a reason for an over-reliance on pads, results from our study also suggest that most nurses are unaware of other treatment or management options that might help nursing staff support patients to self-manage and their UI. Further training on UI, which also highlights how overuse of continence pads can be damaging to patients, could therefore be beneficial and improve patient care.

Additional barriers to assessing UI therefore leading to under-reporting were also identified. Nurses reported that some women who experienced UI tried to hide it or didn’t want to ask for help when experiencing a UI event/episode. This feeds into the culture of UI being a taboo subject and embarrassing to talk about and may present as a barrier to both nurses and women declaring UI. Despite UI having a significant impact on women’s lives the condition is dismissed, ignored and so common it is considered ‘normal’.^29^ This is evident in this study because the difference in reported prevalence in secondary settings compared with community prevalence is stark.

### Limitations

One limitation of this study is that for practical reasons the nurse interviews that were conducted for phase 2 were conducted with nurses from a different NHS hospital than where the EPCR data were collected for phase 1. While these hospitals were both NHS teaching hospitals based in the north of England and comparable in size the fact that they were different hospitals should be taken into consideration when drawing conclusions about the reasons for under-reporting of UI on EPRC as it is possible that nurses experiences of conducting nursing assessments and knowledge may differ or not reflect practices at the hospital providing electronic data.

Another limitation of this study is that separate data regarding urgency UI, stress UI or mixed UI was not available. This was due to the nature of the nursing assessment which is conducted and recorded within EPCR.

## Conclusion

Overall results of this study suggest that UI is being drastically underreported within EPCR. Qualitative results demonstrate that nurses may be under-reporting based on misconceptions about UI. Nurses also demonstrated limited knowledge of UI and potential interventions and had limited training on continence care. Given that older women who experience UI during hospital admission are at increased risk of hospital-acquired harm such as pressure ulcers, falls, urinary tract infections, and increased likelihood of sepsis for those catheterised, it is imperative that women are provided with quality continence care to prevent hospital-acquired harm. Our results highlight the need for nurse training on continence and suggest that improvements could be made in terms of care around continence for older women including better clinical assessment to identify the specific causes and additional nuanced interventions to address continence issues.

## Supplemental Material

sj-docx-1-whe-10.1177_17455057241295607 – Supplemental material for Exploring urinary incontinence in hospitalised older women: A mixed methods investigation of prevalence and nurse perspectives Supplemental material, sj-docx-1-whe-10.1177_17455057241295607 for Exploring urinary incontinence in hospitalised older women: A mixed methods investigation of prevalence and nurse perspectives by Isobel McMillan, Liz Doxford-Hook, Julie Wood, Yu Fu, Linda McGowan and Heather Iles-Smith in Women’s Health

## Declarations

**Ethics approval and consent to participate:** University of Salford Ethics and NHS Health Research Authority approval for the research was granted (IRAS project ID: 303118). Written informed consent was gained from all interview participants.

**Consent for publication:** Not applicable.

**Availability of data and materials:** Not applicable.
