# Supplementary material for: Exploring urinary incontinence in hospitalised older women: A mixed methods investigation of prevalence and nurse perspectives
Source: Womens Health (Lond). 2024 Dec 15;20:17455057241295607. doi: 10.1177/17455057241295607 (PMC11647998; doi:10.1177/17455057241295607)
Supplement: sj-docx-1-whe-10.1177_17455057241295607 – Supplemental material for Exploring urinary incontinence in hospitalised older women: A mixed methods investigation of prevalence and nurse perspectives [file sj-docx-1-whe-10.1177_17455057241295607.docx]

**Good Reporting of A Mixed Methods Study (GRAMMS)**

| **Guideline** | **Section: page** |
| --- | --- |
| Describe the justification for using a mixed methods approach to the research question | Method: page 3-6 |
| Describe the design in terms of the purpose, priority and sequence of methods | Method: page 3 |
| Describe each method in terms of sampling, data collection and analysis | Method: page 3-6 |
| Describe where integration has occurred, how it has occurred and who has participated in it | Method (Phase 3: Integration and interpretation of data): page 6 |
| Describe any limitation of one method associated with the present of the other method | Discussion (limitations): page 21 |
| Describe any insights gained from mixing or integrating methods | Results (Phase 3: Integration and interpretation of data): Page 19  Discussion: page 19-21 |

*O'Cathain A, Murphy E, Nicholl J. The quality of mixed methods studies in health services research. J Health Serv Res Policy. 2008;13(2):92-98.*
